# Adaptive Radiation for Lung Cancer

**DOI:** 10.1155/2011/898391

**Published:** 2010-08-04

**Authors:** Daniel R. Gomez, Joe Y. Chang

**Affiliations:** Department of Radiation Oncology, The University of Texas M. D. Anderson Cancer Center, 1515 Holcombe Boulevord, Houston, TX 77030, USA

## Abstract

The challenges of lung cancer radiotherapy are intra/inter-fraction tumor/organ anatomy/motion changes and the need to spare surrounding critical structures. Evolving radiotherapy technologies, such as four-dimensional (4D) image-based motion management, daily on-board imaging and adaptive radiotherapy based on volumetric images over the course of radiotherapy, have enabled us to deliver higher dose to target while minimizing normal tissue toxicities. The image-guided radiotherapy adapted to changes of motion and anatomy has made the radiotherapy more precise and allowed ablative dose delivered to the target using novel treatment approaches such as intensity-modulated radiation therapy, stereotactic body radiation therapy, and proton therapy in lung cancer, techniques used to be considered very sensitive to motion change. Future clinical trials using real time tracking and biological adaptive radiotherapy based on functional images are proposed.

## 1. Introduction

The treatment of lung cancer with radiation has undergone significant improvements over the past decade. These improvements can be grouped under three different categories. First, it is possible to better delineate the target volume through the advancement of imaging such as positron emission tomography (PET) fusion [1, 2]. Second, there has been an increase in the utilization of radiation planning and delivery systems such as intensity-modulated radiation therapy (IMRT) [3], stereotactic body radiation therapy (SBRT) [4], and proton therapy [5] that allow more conformal delivery of radiation to achieve dose escalation while minimizing toxicity to normal structures [6, 7]. Finally, the advancement of imaging modalities available during the planning and delivery process has made it feasible to adapt the target volume, both within a given fraction of treatment and between fractions, for such factors as internal motion, tumor response, anatomical changes, and weight loss. With the availability of 4D CT—(four-dimensional computerized tomography)—based radiotherapy planning and on-board imaging (OBI), accurate positioning of the target using daily image guidance may result in many advantages, such as a decreased probability of target miss, smaller setup margins, and less normal tissue exposed to high radiation doses. In addition, novel treatment adaptation algorithms can provide more effective modification of treatment plans to adapt to the changes in a patient's anatomy and organ motion during and/or between (intra- and/or inter-) treatment fractions. This latter category of innovations has been termed “adaptive radiotherapy,” and will be the focus of this paper.

In this examination of adaptive radiation in the setting of lung cancer, we will assess the impact of this development in SBRT, conventionally fractionated IMRT, and proton therapy. We will first define key terms in adaptive radiation, and will then review prior studies assessing the magnitude of intrafractional changes, which we define as changes within a given fraction of radiation, from setup to delivery. This discussion will be followed by the effect of these changes on delivered dose and specific planning techniques that have been utilized to decrease this variation, specifically cone beam computed tomography (CBCT) and respiratory gating. We will then focus on interfraction changes that occur during the length of treatment (over a period of several weeks), such as changes in tumor mobility, tumor volume, anatomy, and body weight, and examine specific studies that assess the impact of adaptive planning on improving dose distributions in these setting. Finally, we will appraise future directions of adaptive radiation, including selective dose escalation through the visualization of nonresponsive regions and internal tracking to monitor intrafraction motion in real-time, both of which may be able to allow for further sparing of critical structures while maintaining accurate delivery for treatment of this aggressive disease.

## 2. Important Definitions in Adaptive Radiation for Lung Cancer

As adapted from the International Commission on Radiation Units (ICRU) Report no. 50: [8].

### 2.1. GTV (Gross Tumor Volume)

 Tumor visible by any imaging modality, to include both the primary tumor and any involved lymph nodes. Lymph nodes greater than 1 cm in size in the shortest axis are generally considered positive for disease [9], but functional imaging such as PET scanning is critical for target delineation [10, 11].

### 2.2. CTV (Clinical Target Volume)

The anatomically defined region at risk for microscopic disease. This region cannot be visualized as a discrete structure with radiographic imaging, and the extent has been defined by surgical series with pathologic correlates, as well as autopsy series. As defined by Giraud et al., based on NSCLC surgical specimens, an appropriate GTV to CTV margin for adenocarcinoma is 8 mm, and for squamous cell carcinoma, 6 mm [12]. Another study found that in stage I adenocarcinoma, a margin of 9 mm is sufficient to cover 90% of disease [13]. In terms of mediastinal disease, a GTV to CTV margin has not been rigorously analyzed, but based on an abstract presented at the 2006 meeting of the American Society for Therapeutic Radiology and Oncology that found a maximal microscopic extension of lymph nodes is between 0.5 and 8.9 mm [14], we routinely use margins of 8 mm for nodal disease.

### 2.3. ITV (Internal Target Volume)

 CTV with a margin to account for organ motion; or, in other words, changes in the size, shape, and position of the target during treatment [15]. Delineating the ITV from 4D CT images involves assessing the target volume (CTV) on expiratory-phase images and then registering the outline to the images from other phases to create a union of target contours enclosing all possible positions of the target. An alternative method is to create a maximum intensity projection (MIP) image by combining data from the multiple CT data sets with data from the whole-breath cycle and modifying the ITV by visually verifying the target volume throughout the breathing phases (typically 10). In this process, attention should be paid to irregular breathing and breathing pattern variations during each treatment session and over the entire treatment course, as well as to the effects of these irregularities on the ITV margin. 

Because it is often more straightforward to delineate the boundaries of the gross tumor volume with motion on 4D CT image data sets as opposed to the clinical target volume, we previously proposed the concept of the *internal gross tumor volume* (iGTV), which envelops the GTV motion throughout the whole-breath cycle [16]. In this process, rather than delineating the GTV, expanding to the CTV, and then adding the ITV, the GTV is contoured, motion is assessed as outlined above (i.e., through the MIP images or through all breathing phases), and this target is expanded to the iGTV. Then, the iGTV is expanded to the ITV (ITV = iGTV + CTV). This latter method is what is commonly utilized at our institution to account for organ motion in the treatment of NSCLC.

### 2.4. PTV (Planning Target Volume)

 CTV plus a margin to account for both organ motion and daily setup. Or, the ITV with a margin to account for daily setup. The application and revision of margins for the PTV and ITV will be discussed in detail below.

## 3. The Role of Adaptive Radiotherapy in Intrafraction Adaptive Planning

### 3.1. Quantitating Intrafractional Tumor Motion

Several studies have attempted to quantitate intrafraction tumor motion with radiation therapy. Bissonnette et al. examined CBCT images during each fraction of 18 patients receiving SBRT for medically inoperable Stage I nonsmall cell lung cancer (NSCLC). The CBCT images were performed at the beginning, midpoint, and end of each fraction to determine differences in tumor motion amplitude. The authors found that at a mean time of 35 minutes (from the beginning of each treatment to the end), the mean change in tumor amplitude was 0.4, 1.0, and 0.4 mm in the medial-lateral (ML), superior-inferior (SI), and anterior-posterior (AP) directions, respectively. These values were not statistically significantly different when compared to the initial respiratory correlated (4D) CT scan, other than in patients in which abdominal compression was used, in which longer times on the couch were hypothesized to increase these differences [17]. In another study, Michalski et al. compared the amount of motion between the three aforementioned directional axes (ML, SI, and AP) in 23 patients undergoing 4D CT scans. The authors found that the largest intrafractional extent of motion was in the SI direction, with the largest range being 3.59 cm [18]. A similar study from our institution also assessed respiration-induced tumor motion during radiation for lung cancer. This study by Liu et al. assessed 166 tumors in 152 lung cancer patients, 57% of whom had stage III or IV disease. The authors also found that the largest axis of motion was in the SI direction, and that 39% of tumors moved >0.5 cm in this direction, compared to 1.8% and 5.4% in the ML and AP directions, respectively. Tumor motion was also found to be correlated with the amount of diaphragm motion, the SI location of the tumor, and the size of the GTV, with smaller tumors exhibiting a greater degree of intrafractional motion [19]. A study by Thomas et al. demonstrated that mediastinal nodal regions also move substantially with the respiratory cycle, generally posteriorly and superiorly with exhalation, and that inferior nodal stations exhibit a greater degree of motion, such that the same issues with respiratory variation in parenchymal tumors can also be applied to mediastinal disease [20]. 

Thus, it can be concluded that: (1) inferior tumors move more than superior tumors, (2) the largest axis of motion is in the SI direction, and (3) mediastinal lymph nodes are also subject to a significant degree of tumor motion.

### 3.2. On-Board Imaging in Treatment for NSCLC

There are several methods to detect setup error and intrafractional variation during radiation therapy for NSCLC.

#### 3.2.1. Electronic 2D Imaging with Radiographs

 In this technique, two-dimensional images are composed in the treatment position, and the patient is set up on patient anatomical landmarks such as bony anatomy. Historically, megavoltage (MV) imaging had been used, but the images produced with this technique were generally of poor image quality and exposed the patient to high radiation doses. In the past decade, the utilization of kilovoltage (kV) imaging has increased, which has improved image quality and decreased radiation exposure.

#### 3.2.2. Kilovoltage or Megavoltage Cone-Beam CT Scanning

**Figure 1:**
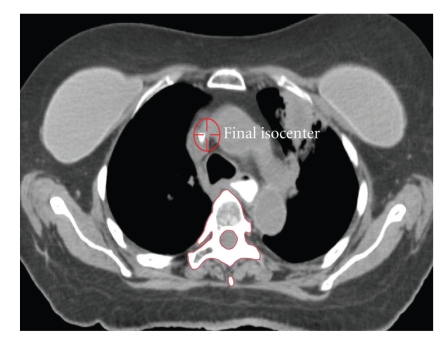
Volumetric on-board Kilovoltage cone-beam CT imaging.

 In this method, a CT image is reconstructed on the treatment table from a set of projection images acquired at multiple angles around the patient. Image reconstruction with this modality differs from conventional CT scans in that rather than a linear array of detectors being back projected to construct a 2D slice, a “detector array” (e.g., a flat-panel portal imager) is used to reconstruct a 3D data set [21]. (Figure 1) It follows that the CT images that are a result of this process are theoretically superior in improving intrafractional error as compared to portal images, and multiple studies have exhibited this improvement, particularly in SBRT where longer treatment times can lead to greater instability and less reproducibility of the setup process. 

### 3.3. Setup Errors as a Cause of Intrafractional Variation and the Role of On-Board Imaging

Intrafraction treatment variation has two components in lung cancer: uncertainties in patient positioning and variations in internal motion. Nelson et al. examined the effect of lung tumor motion and setup uncertainties using implanted fiducial markers. The authors found that systematic and random uncertainties ranged between 4 and 6 mm in all three directions [22]. With daily portal imaging, the authors found in a subsequent study that alignment based on implanted fiducials reduced systematic errors in the left-right and superior-inferior direction each by 3 mm [23]. As a result, daily portal imaging is often utilized to decrease setup error in conventionally fractionated regimens, with a daily setup error of up to 5 mm. 

Borst et al. compared CBCT setup with portal imaging in daily setup for 62 patients in 524 scans with NSCLC, and found that CBCT reduced the setup error to less than 5 mm, from 51% of patients with setup errors more than 5 mm to 2% with CBCT [24]. Bissonnette et al. assessed the accuracy of CBCT in RT for lung cancer in patients receiving both SBRT for early-stage malignancies and in those patients undergoing conventionally fractionated treatment for locally advanced disease. The couch position was adjusted for discrepancies greater than 3 mm between the initial setup and treatment images. The accuracy of this adjustment was then verified with a second CBCT. Without CBCT adjustments, positioning errors were found to exceed 5 mm in approximately 55% of patients. However, CBCT decreased this error, such that systematic and random setup margin was within 3 mm for 82% of fractions in the SBRT group, and between 76% and 84% of the patients in the conventionally fractionated group [25]. Similar findings were published by Grills et al., who found that CBCT image guidance significantly decreased setup margins in SBRT, with calculated precorrection population margins of 9–13 mm and 10–14 mm with a stereotactic body frame and alpha cradle, respectively, while these same margins were 1-2 mm and 2-3 mm postcorrection, and 2–4 mm and 2–5 mm postreatment, respectively [26]. The conclusion of these studies is that consistent CBCT imaging can decrease the amount of systematic and random error on a daily basis (to within 3 mm), and thus decrease intrafraction variation.

### 3.4. Methods of Breathing Control

Lung tumors differ from many other treatment sites in that internal motion can account for large variations in tumor position, and as a result, dosimetry. Several studies have exhibited this finding. Mechalakos et al. examined treatment plans for 12 patients receiving radiation therapy for NSCLC. The authors found that the dose to 95% of the gross tumor volume (GTV), also known as the D95, changed on an average of only 1.4% when normal breathing effects were incorporated. However, with “heavy breathers,” the D95 changed almost 10%. Therefore, the authors concluded that while the chance of a 10% or greater decrease in D95 was less than 4%, patients with a large degree of respiratory motion could have significant effects, and thus these patients should be identified [27]. A study from the University Hospital Rotterdam in the Netherlands found that with a GTV to planning target volume (PTV) margin of 1.5 cm, approximately 11% of the tumor was not covered in mobile tumors. Engelsman et al. found that when combined with setup error, respiratory motion reduced the tumor control probability (TCP) by almost 9% (from 50% to 42%) in patients receiving conventionally fractionated radiation to 70 Gy in lung tumors [28]. 

There are several methods in which tumor motion can be taken into account with the delivery of radiation therapy and which are utilized at our institution. First, breathing can be monitored during simulation and the iGTV/ITV can then be added as a margin to ensure that the tumor is treated adequately throughout all phases of the respiratory cycles as outlined above (e.g., if the tumor is noted to move 1.5 cm during treatment, then a 1.5 cm margin, termed the ITV/iGTV, is added to account for this respiratory motion.). This strategy is often denoted the “free-breathing technique.” If 4D CT imaging is not available at the institution, then an alternative method to account for respiratory motion is through the use of breath-hold spiral CT simulation. In this method, the patient is instructed to hold his breath during simulation, both at end inspiration and expiration. Images are then taken at end-inspiration and end-expiration, such that an ITV can be generated by combining the two CTVs from the inspiratory and expiratory scans. 

A disadvantage to the free-breathing method is that in tumors that have a large magnitude of motion, the amount of normal tissue that is treated can be relatively large, since radiation is being delivered throughout the breathing cycle. Therefore, an alternative method of radiation delivery is to instruct the patient to hold their breath during treatment and activate the radiation while the tumor is in this full inspiratory, fixed position. The disadvantage to this technique (often termed deep inspiratory breath hold, or DISB) is that it requires full cooperation of the patient, in that the patient will need to hold their breath for 15 seconds or longer. As an alternative to this method, radiation could be delivered in either a relaxed inspiratory position or in expiration, which is often more reproducible than DISB.

**Figure 2:**
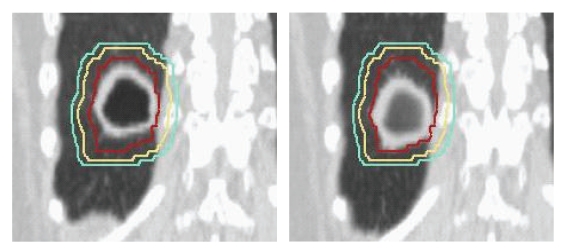
Motion of a tumor in the inferior portion of the lung.

In patients that are not able to comply with any of these instructions, a third method of delivering radiation in tumors that have a great deal of motion is through a ventilatory-gated approach, during which the radiation beam is coordinated with the respiratory cycle through the placement of externally placed fiducial markers. Or, in other words, the radiation is only delivered at certain phases, most typically full expiration [29]. This type of therapy is generally most useful for tumors less than 5 cm in diameter and for a tumor motion of greater than 1 cm. Figure 2 demonstrates the motion of a tumor in the inferior portion of the lung. It is evident that with timed radiation delivery, a great deal of normal tissue could be spared by reducing the treatment margins.

### 3.5. Dosimetric Advantages of Breathing Control in NSCLC

Many studies have shown that accounting for breathing motion can improve dosimetric parameters. Vlachaki et al. assessed 10 patients with lung tumors to determine the effect of respiratory gating on dosimetry. Gated images were acquired at full inspiration, full expiration, and at each quartile of respiratory movement. The authors found that gating led to higher minimum target volume doses, and that the V20 was reduced from 35% to 26% for gated plans. The mean lung, heart, and esophageal doses were also lower with gated plans. The authors concluded that gating could improve the dose to normal structures while maintaining target tissue coverage [30]. Further studies have corroborated these findings. Underberg et al. examined 31 patients simulated with 4D CT scans and compared the dose to normal tissue in three different techniques to account for breathing motion in target delineation: (1) standard, population-based margins to account for internal motion, (2) the generation of an ITV based on tumor mobility in three consecutive phases, and (3) a PTV generated from respiratory gating. The authors concluded that utilizing “standard population-based” margins led to unnecessary normal tissue irradiation, and that the risk was best minimized if gating was utilized [31]. A recent study from Fox Chase Cancer Center arrived at a similar conclusion, in that 4D CT-based treatment planning maintains target coverage while reducing normal tissue dose [32]. 

 It is important to note that the intrafractional adaptive radiotherapy techniques of CBCT and breathing control are not mutually exclusive, but in contrast should be utilized together to optimize the therapeutic ratio, as demonstrated by several studies. Koch et al. found that the relationship between internal lung motion and skin fiducial motion is complex and unpredictable, and that the AP motion of tumors correlated poorly with skin surface markers. They also found that there is significant intersubject variability [33]. In a follow-up study, Liu et al. found that this variability (and in turn, the target volume margins) could be substantially reduced with the use of respiratory gating techniques [34]. And in a study by Nelson et al., the authors assessed the margins necessary to account for uncertainties in tumor position with respiratory gating, image-guided patient setup, neither, or both. The authors found that utilizing both methods simultaneously allowed for the greatest reduction in margins that completely encompassed the tumor, and therefore concluded that when respiratory motion management is used, it should be used “in conjunction with image-guided patient setup in order to reduce the overall treatment margin effectively” [35].

### 3.6. Real-Time Tracking in NSCLC

Another delivery system of increasingly widespread use is Cyberknife therapy, which utilizes a stereotactic guidance system designed primarily for radiosurgery in multiple organ systems. The premise behind this system is that the linear accelerator is mounted on a robotic arm, which can then track the target during treatment. Cyberknife has been reported in single institution studies in the setting of SBRT for lung cancers. For example, Le et al. reported 32 patients with lung tumors treated in a dose escalation study using single fractions, and found a 1-year local control rate of 91% for doses greater than 20 Gy and at doses less than 25 Gy there was no significant toxicity [36]. Brown et al. assessed the efficacy of this technique in peripherally located stage I nonsmall cell lung cancers. In a cohort of 31 patients, the authors reported no grade 3 or higher toxicities and 1-year local control rates of 93.2% [37]. Further studies on this technique will continue to define its role in adaptive radiotherapy and the definitive treatment of NSCLC. 

More recently, Novalis Tx provides stereo X-ray targeting and adaptive gating using ExacTrac X-ray 6D and snap verification system. Videtic et al. reported 94.4% local control with minimal toxicities with 50 Gy delivered in 5 fractions using the Novalis/Brain LAB system. All of these novel systems may provide an optimal treatment for clinical challenging cases such as patients with poor lung function and/or lesions close to critical structures [38]. 

## 4. The Role of Adaptive Radiotherapy in Interfractional Changes

Several factors change during the course of radiation can affect target and normal tissue dose. These factors include but are not limited to changes in tumor size, alterations in tissue anatomy, variations in respiratory patterns, and reductions in patient weight. In this setting, adaptive radiotherapy refers to repeat assessment of the target volume, either through repeat CBCT, 4D imaging, or both. 

### 4.1. Changes in Tissue Anatomy, Respiratory Patterns, and Weight with IMRT and 3D-Conformal Therapy

Multiple studies have been performed examining causative factors of interfractional changes and the effect of these variations on dosimetry throughout treatment. Redmond at al. examined 10 patients to determine whether tumor excursion due to respiratory motion was stable when comparing images acquired at the time of simulation with those during treatment. The authors found that while there was interfraction consistency in tumor excursion during treatment, the relationship between the GTV and other anatomic structures between respiratory changes varied with rescanning. The authors therefore expressed caution in relying on “surrogate anatomic markers” to assess tumor motion throughout treatment [39]. Bosmans et al. assessed 23 patients with locally advanced NSCLC who underwent CT-PET and respiration-correlated imaging prior to treatment, and repeated at the first and second weeks after the start of radiation. The authors observed that while changes in tumor motion were relatively small, there was a great deal of variation in tumor size during therapy. These changes ranged from an increase in greater than 30% to a decrease of the same magnitude. The authors concluded that the changes in tumor size warranted assessment for replanning during the course of therapy [40]. Van Zwienen et al. found that clinically evident regression occurred in approximately 40% of patients undergoing definitive treatment for NSCLC, with approximately 10% of patients undergoing >25% reduction in size by week three and in 24 out of 114 patients by week four. Larger reductions were also associated with reduction atelectasis [41]. A study from Johns Hopkins University supported this finding of large variations in the GTV, and thus also recommended an adaptive approach in conventionally fractionated patients [42]. 

**Figure 3:**
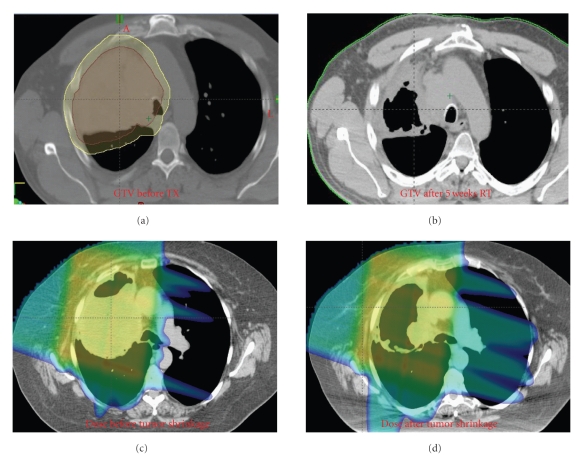
Impact of tumor shrinkage in proton isodose distribution over 7 weeks of treatment.

Britton et al. analyzed the effect of gross tumor volume regression and motion changes during the course of radiation therapy in locally advanced NSCLC at MD Anderson Cancer Center. The authors found that in 8 patients with weekly 4DCT data sets, the tumor volume was reduced by a range of 15–71%. There was also noted to be increased tumor mobility in the SI and AP directions throughout treatment, without any clear trends in tumor motion [43]. In a follow-up study at the same institution, the authors found that the dose to 95% of the PTV and ITV with weekly CT scans changed by approximately 12% and 2.5%, respectively. While the lung V20 and mean lung dose only increased by a mean of 3.1% and 2.2%, respectively, the spinal cord dose changed by an average of 34.3% [44]. These studies together imply that continued assessment of the target volume is recommended and is essential in tumors that lie near the spinal cord. Figure 3 demonstrates the significant reduction in size of a tumor throughout treatment, and the large effect that this reduction has on the dose distribution. 

A study from Denmark demonstrated that anatomical and motion changes persist even with respiratory gating. In a study by Juher-Nottrup et al., ten patients receiving 60 Gy in 2 Gy fractions underwent serial 4D CT scans during treatment. The authors found that the interfractional overlap of lung tumors was only 80%–87% when bony landmarks were used with a gating technique, and that this overlap decreased to 70%–76% when skin tattoos were present. With mediastinal tumors, the overlap was 60%–65% and 41%–47%, respectively. It can therefore be concluded that gating does not preclude interfractional adaptive planning in patients with lung or mediastinal tumors [45]. 

Weight loss can be a factor in changing anatomy and in turn the dose to target volume, such as in the case of a patient that loses a great deal of weight that causes the effective beam path to change. Some investigators have questioned whether weight loss in itself can be a cause of setup error during the course of treatment due to factors such as changes in the position of skin marks. Johansen et al. attempted to answer this question by evaluating the relationship of interfractional setup errors with body mass index and weight loss. The authors assessed 34 head and neck cancer patients and 20 lung cancer patients who received serial CBCT images to evaluate whether there was a change in 3D position between the initial CBCT scans and those from the 10th and 20th treatment session. The study did not find a statistically significant correlation between setup error and either patient body mass index or weight loss [46]. Therefore, while it is still recommended that replanning is performed on the basis of weight changes during treatment, interfractional changes as a result of weight loss may be more a function of subsequent anatomical variations rather than true setup error.

### 4.2. Interfractional Adaptive Planning in SBRT

Similar studies as above quantitating interfractional dosimetric error have been performed in patients being treated with SBRT. Matsugi et al. examined 4D CT scans for 8 patients being treated with SBRT, to measure interfraction variations in position and the size of target volumes with this technique. In contrast to a conventionally fractionated treatment which is several weeks in duration, the authors found that the size of the GTV did not change significantly during treatment and that variations in motion range and position were also small [47]. Similar conclusions were made by Haasbeek et al., who found that the dosimetric consequences of interfraction adaptive planning were small [48]. However, the target volume is usually small in SBRT and a large fraction size is delivered in less than 5 fractions; therefore, missing a small volume of target even just in one fraction can cause significant underdosing of the tumor and/or overdosing surrounding normal tissues. We therefore reiterate that we recommend daily volumetric verification for target coverage in SBRT.

### 4.3. Interfractional Adaptive Planning in Proton Therapy

With the advent of proton therapy, investigators have recently begun to examine the effect of interfractional motion and anatomic changes on dose distribution with this technique. This issue is particularly important with protons because the impact of motion and anatomy changes is more significant in proton compared with photon (Figure 3). Hui and Chang et al. acquired weekly 4D-CT scans on 8 patients with locally advanced NSCLC treated with IMRT. A conformal passive scattering plan was generated for each patient and compared with IMRT plan over 7 weeks of radiotherapy. The authors found that normal tissue doses were increased and CTV coverage was significantly compromised in one patient (with a decrease in approximately 8%) with proton therapy but much less significantly in the IMRT plan. The authors concluded that proton therapy is more sensitive to motion and anatomy changes compared with photon and interfractional adaptive planning and is indicated in select patients [49]. 

### 4.4. Guidelines for Adaptive Planning in Nonsmall Cell Lung Cancer

At our institution, all patients undergo a 4D CT simulation to assess for internal motion. Patients are immobilized with an upper body cradleand T-bar, which has a daily setup uncertainty of approximately 7 mm. At the time of simulation, patients are evaluated for breathing patterns and tumor motion. In patients whose tumor motion is less than 1 cm, a “free-breathing” technique is typically used, with the creation of an ITV or iGTV and radiation treatment delivery in all phases of the breathing cycle. If the target volume moves more than 1 cm and the patient can breathe reproducibly, then radiation is either timed with certain phases of the breathing cycle while the patient breathes freely (ventilatory gated technique), or the patient is instructed to hold their breath for at least 15 seconds while radiation is delivered at deep inspiration (DISB). We utilize visual and/or audio feedback guidance for patients who can comply with these devices. 

Patients undergoing standard fractionated regimens are initially set up daily with kV imaging for verification, which reduces the setup margin to 5 mm. All patients are assessed for concordance between bony/skin landmarks and tumor setup. If it is found that these setup parameters are discordant, or that the tumor is changing rapidly, CBCT is employed, which reduces the ITV to PTV margin to 3 mm. All patients receiving SBRT undergo volumetric verification of set up and motion such as CBCT prior to each treatment. 

Repeat 4D simulations are performed selectively at the physician's discretion in patients with NSCLC undergoing conventional fractionation for 6-7 weeks and are routinely performed in patients with small cell lung carcinoma undergoing hyperfractionated (twice a day) or dose escalated/accelerated radiotherapy for 3 to 7 weeks. Adaptive 4D replanning is performed if motion/anatomy changes may change the target coverage and/or increase dose to surrounding critical structures. Interfractional adaptive 4D planning is not routinely utilized on patients undergoing SBRT (4-10 fractions), as these patients receive daily volumetric imaging. 

It is notable that it is still unclear if target volume reductions are warranted in the scenario of GTV shrinkage during the course of radiotherapy [50–52]. Some physicians advocate an approach in which the target volume remains constant due to concerns for residual microscopic disease. To address these concerns, one option is to deliver at least 50 Gy, the standard dose for microscopic disease, to the original target volume, and then to boost the reduced volume to the full dose. Well-designed studies addressing this issue are needed.

## 5. Future Directions in Adaptive Radiotherapy

New technologies are evolving to improve adaptive radiation therapy, such that the high dose region is focused on the target while sparing critical normal tissues. Treatment planning based on 4D CT images and on-board image-guided adaptive treatment delivery assists the radiation oncologist in tracking tumor motion and targeting the tumor precisely. 

As advancements in technology have allowed for both intra- and interfraction replanning, investigators have begun to assess whether or not the response of tumor during treatment can lead to changes in total dose to portions of the target volume based on the magnitude of tumor response to the initial phase of treatment (image-guided adaptive treatment). This strategy is particularly relevant because several studies have shown that dose escalation in lung cancer improves local control [53]. Furthermore, prior studies have demonstrated that regions with high SUVs and hypoxic areas are more likely to exhibit local failure when treated with radiation [54, 55]. The information thus suggests that the radioresistance and propensity for distant metastasis in gross disease varies based on factors other than histologic type, stage, and tumor grade. It is in this realm that adaptive dose escalation may have a role in addition to the utility of IMRT dose painting.

Feng et al. performed a pilot study in which 14 patients with NSCLC underwent repeat PET-CT scans prior to the start of radiation therapy and mid-way through treatment. Boost fields were then designed based on residual PET avidity. In this dosimetric study, the authors found that this method allowed for a mean dose escalation of 58 Gy or a reduction in normal tissue complication probability (NTCP) of up to 3% in patients with a reduction in tumor size [56]. In a similar study, Gillham et al. performed an additional PET-CT scan in week 5 or 6 of radiotherapy for patients being treated with localized inoperable NSCLC to 66 Gy in 2 Gy fractions. The authors found that there was a median PTV reduction of 20%, and that in this dosimetric study, dose escalation to 78 Gy was feasible in four out of ten patients without exceeding normal tissue constraints [57]. Future studies will determine whether this technique of adaptive dose escalation is feasible from a clinical standpoint without increasing toxicity and while maintaining tumor control.

An additional technologic advancement in the context of adaptive radiotherapy is the development of “real-time” tracking of tumor motion so that the treatment can be actively and variably adapted in concordance with intrafractional changes. In addition to Cyberknife, which has been used clinically as discussed above, another commercially available system is the Calypso Medical three- or four-dimensional electromagnetic tracking system. The system has undergone initial assessment in a wide variety of tumor sites and several studies have been published describing the technique. For example, Smith et al. compared dosimetric results with and without real-time tracking. The authors found that the dose profiles were comparable with an idealized gating algorithm while minimizing the uncertainties inherent in the use of anatomical surrogates for target location, with a high level of efficiency [58]. In a follow-up study, the same authors proposed a method for linear accelerator gating with wireless internal fiducial markers without the requirement for ionizing radiation for imaging, leading to improvements in the dose distribution [58]. 

Adaptive radiotherapy may improve tumor control in lung cancer by reducing target misses, escalating target dose, and minimizing side effects by avoiding critical structures over the course of radiotherapy. More prospective studies are needed to implement these technologies and validate their efficacies.
